# Photodynamic therapy in head and neck squamous cell carcinoma: immunomodulation and stromal targeting of cancer-associated fibroblasts

**DOI:** 10.1016/j.tranon.2026.102910

**Published:** 2026-07-09

**Authors:** Julia Federspiel, Federico Ferro, Jozsef Dudas, Anna Katharina Stenzl, Benedikt Gabriel Hofauer

**Affiliations:** Department of Otorhinolaryngology and Head and Neck Surgery, Medical University of Innsbruck, A-6020 Innsbruck, Austria

**Keywords:** Photodynamic therapy, Nanomedicine, Immunogenic cell death, Cancer-associated fibroblasts, Head and neck squamous cell carcinoma

## Abstract

Head and neck squamous cell carcinoma (HNSCC) remains challenging due to its complex tumor microenvironment (TME) and resistance to conventional treatments. Within this context, cancer-associated fibroblasts (CAFs) play a central role in stromal remodelling and immune evasion. Photodynamic therapy (PDT) employs a photosensitizer (PS) activated by light to generate reactive oxygen species (ROS), inducing direct cytotoxicity and immunogenic cell death (ICD). Conventional PS achieve high local control in early-stage or superficial HNSCC but are limited by hypoxia, stromal stiffness, and restricted light penetration. Nanoparticle-based PS improve tumor and stromal targeting, modulate CAF activity, normalize the extracellular matrix (ECM), and facilitate immune infiltration. PDT-induced ICD activates dendritic cells (DCs) and primes CD8⁺ T cells, linking local tumor killing with systemic immune activation. Despite limited clinical evidence, future translational studies are warranted to investigate CAF-targeted PDT combined with standard therapies to remodel the immunosuppressive TME and improve clinical outcomes in HNSCC.

## Highlights

• PDT induces immunogenic cell death and anti-tumor immunity in HNSCC.
• CAFs drive stromal resistance to PDT.
• CAF-targeted PDT remodels the TME and enhances photosensitizer delivery.
• Combining PDT with immunotherapy may enhance therapeutic efficacy.

## Graphical abstract

**Figure:**
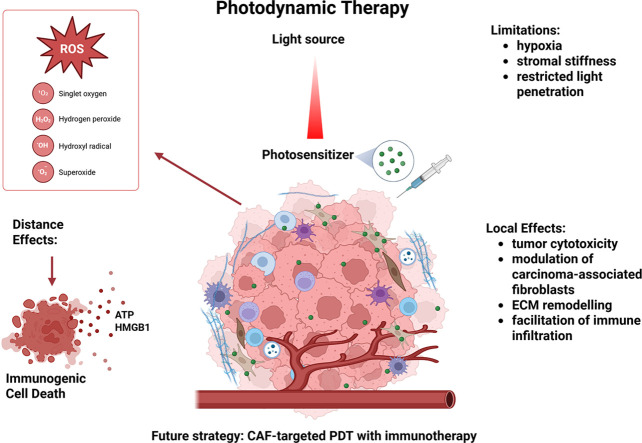

## Introduction

HNSCC represents a heterogeneous malignancy characterized by a highly dynamic TME. It comprises diverse stromal and immune cell populations, ECM components, and complex biochemical gradients that collectively regulate tumor initiation, progression, and immune evasion. Within the TME, CAFs constitute a dominant and functionally diverse stromal population that plays a central role in modulating tumor behavior [1]. CAFs exhibit pronounced functional plasticity shaped by tumor context. In HNSCC, their activities are predominantly pro-tumorigenic and include ECM remodelling, paracrine signaling, and the release of CAF-derived exosomes (CAF-Exos), which facilitate stromal-tumor-immune crosstalk [2,3].

Through dynamic interactions with malignant and immune cells, CAFs contribute to the establishment of an immunosuppressive TME. This is achieved by secretion of transforming growth factor-β (TGF-β), interleukin-6 (IL-6), and C-X-C motif chemokine ligand 12 (CXCL12) that promotes recruitment of regulatory T cells and polarization of macrophages toward an M2-like phenotype [3]. These M2 macrophages can suppress cytotoxic anti-tumor immunity [4,5]. CAF-Exos promote immune evasion by upregulating the immune checkpoint ligand PD-L1 on tumor and immune cells [2].

The standard of care in HNSCC is a multidisciplinary approach including surgery, radiochemotherapy, and immunotherapy [6,7]. In addition, PDT has emerged as a minimally invasive approach in selected patients.

PDT is a local tumor treatment modality applied in skin cancer, head and neck cancer (HNC), esophageal cancer, malignant gliomas, and other malignancies. Since its introduction in head and neck oncology in the late 1980s, PDT showed promising responses in recurrent and superficial lesions [8,9]. PDT has been further investigated for local tumor control, particularly in early-stage disease or in patients unsuitable for surgery or radiotherapy [[10], [11], [12]]. Importantly, PDT can provide favorable functional and cosmetic outcomes while preserving surrounding structures and maintaining quality of life [13,14].

**Fig. 1:**
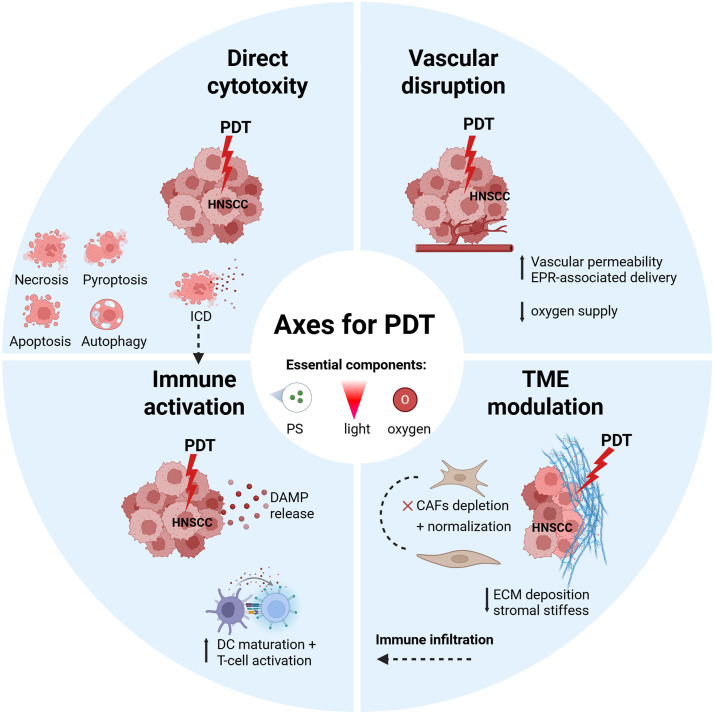
Multifunctional effects of PDT in HNSCC. PDT induces ROS upon light activation, leading to direct tumor cell killing and vascular disruption. Beyond these effects, PDT reshapes the TME by inducing ICD with release of DAMPs, thereby promoting DC maturation and T-cell–mediated antitumor immunity. In addition, PDT targets stromal components, including CAFs, resulting in ECM remodelling and stromal reprogramming. These mechanisms highlight PDT as a multimodal and immunomodulatory therapeutic approach in HNSCC. **Abbreviations:** PDT: photodynamic therapy; ROS: reactive oxygen species; TME: tumor microenvironment; ICD: immunogenic cell death; DAMPs: damage-associated molecular patterns; DCs: dendritic cells; CAFs: cancer-associated fibroblasts; ECM: extracellular matrix; HNSCC: head and neck squamous cell carcinoma. Created in BioRender. Dudas, J. (2026) https://BioRender.com/wg7ozx2 (accessed on 31 May 2026).

**Fig. 2:**
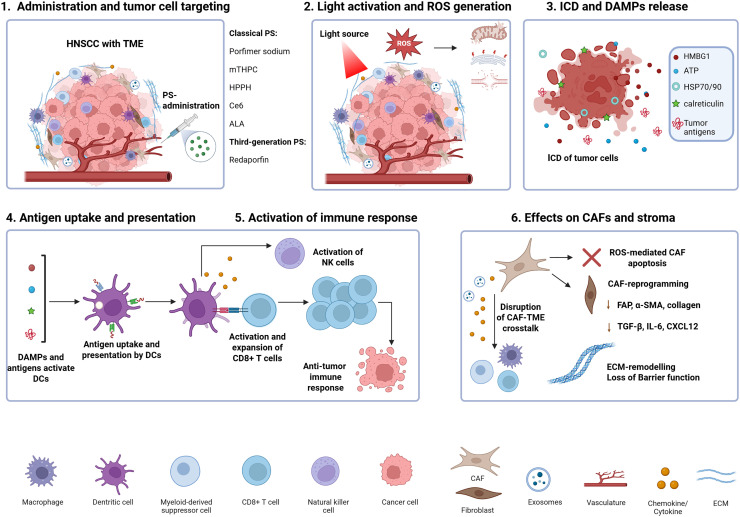
Mechanisms of PDT-mediated immunomodulation and stromal remodelling in HNSCC. (1) PS administration enables preferential accumulation within tumor tissue. (2) Upon irradiation, the activated PS generates ROS, resulting in oxidative damage, mitochondrial cytochrome c release, ER stress, membrane permeabilization, and multiple forms of tumor cell death. (3) ICD is characterized by the release of DAMPs, including HMGB1, ATP, HSP70/90, calreticulin, and tumor-associated antigens. (4) DAMPs and tumor antigens are taken up by DCs, leading to antigen processing and presentation. (5) This activates innate and adaptive immune responses, including NK cells and CD8+ T cell expansion. (6) PDT modulates stromal components by altering the expression and secretion of CAF-derived cytokines, resulting in ECM reorganisation, reduced immunosuppression, and enhanced anti-tumor immunity. The bottom panel summarizes the key players of the TME in HNSCC. Aberrations: PDT: photodynamic therapy; TME: tumor microenvironment; HNSCC: head and neck squamous cell carcinoma; PS: photosensitizer; ROS: reactive oxygen species; ER: endoplasmic reticulum; ECM: extracellular matrix; ICD: immunogenic cell death; DAMPs: damage-associated molecular patterns; HMGB1: high mobility group box 1; ATP: adenosine triphosphate; HSP70/90: heat shock proteins 70 and 90; DCs: dendritic cells; NK cells: natural killer cells; CAFs: cancer-associated fibroblasts. Created in BioRender. Dudas, J. (2026) https://BioRender.com/66e955n (accessed on 31 May 2026).

There are two main approaches to PDT. Flat light sources are used for superficially spreading tumors, while needle and cannula devices can be inserted deep into tissues [15]. PDT relies on three essential components: a PS, light of an appropriate wavelength and molecular oxygen. Upon activation, PDT induces the generation of ROS, leading to direct tumor cell cytotoxicity and vascular damage. In addition PDT reshapes the TME and promotes ICD [16,17] (Fig. 1). PDT-mediated ICD is characterized by the release of tumor antigens, heat shock proteins (HSP) 70/90 and damage-associated molecular patterns (DAMPs), including calreticulin, high mobility group box 1 (HMGB1) and adenosine triphosphate (ATP) [18]. These signals promote DC activation and enhance antigen presentation, thereby linking local tumor destruction to systemic anti-tumor immune responses, as illustrated in Fig. 1 and in greater detail in Fig. 2**.**

The selectivity of PDT arises from the spatial confinement of ROS generation to illuminated tissue and the preferential accumulation of PS within tumor regions. Despite continued advances, PDT remains limited by restricted light penetration depth, tumor hypoxia, and the risk of local recurrence [7]. In addition, the risk of skin and ocular damage post-PDT, due to exposure to indoor or sunlight, further complicates its use in clinical practice [9]. To overcome these limitations, recent strategies aim to target the tumor-specific stroma, including CAFs.

Fibroblast activation protein (FAP) represents an attractive target in this context, as it is highly expressed by CAFs while showing minimal expression in most normal adult tissues [19]. Using PS directed against FAP, PDT allows the delivery of PS to CAFs which are adjacent to tumor cells. This approach can disrupt tumor-supportive microenvironmental functions and co-target tumor cells. FAP-expressing CAFs preferentially accumulate targeted PS through ligand- or antibody-mediated binding in combination with enhanced vascular permeability and high metabolic activity [20].

Emerging preclinical evidence from HNSCC-related and other solid tumor models suggests that CAF-targeted PDT may remodel the tumor stroma and synergize with immunotherapeutic strategies, particularly when combined with nano-based delivery systems [21]. However, direct experimental validation in HNSCC remains limited. Unlike previous review articles, this scoping review focuses on the possible adaptation of PDT to meet the clinical requirements of HNSCC, with a particular focus on tumor and CAF-specific targeting, overcoming the difficulties caused by hypoxia, and the major relevance of activating ICD.

## Material and methods

This scoping review followed the PRISMA Extension for Scoping Reviews (PRISMA ScR) framework to ensure transparent and reproducible study selection. We systematically retrieved literature from PubMed, Web of Science, and Embase, encompassing publications available up to January 2026.

The search strategy combined controlled vocabulary and free-text terms using Boolean operators, including (‘photodynamic therapy’ or ‘PDT’), (‘photosensitizer’), (‘head and neck squamous cell carcinoma’ or ‘HNSCC’ or ‘head and neck cancer’), (‘cancer-associated fibroblasts’ or ‘CAFs’ or ‘tumor microenvironment’), and (‘immune modulation’ or ‘nanodrugs’). Searches were primarily restricted to English-language publications and included human studies as well as in vitro and in vivo models relevant to HNSCC or TME research; one relevant German-language study was also included.

Eligible publications included original research articles and review articles. Original studies constituted the primary evidence base for qualitative synthesis, while review articles were included to provide contextual background and to support interpretation of emerging concepts. Studies were qualitatively synthesized with priority given to those providing mechanistic, translational, or clinically relevant insights. Duplicate publications and studies lacking thematic relevance were excluded.

Reference management and duplicate removal were performed using Zotero (version 8.0.1; Corporation for Digital Scholarship, Fairfax, VA, USA). In total, 192 records were identified, and 34 duplicates were removed, leaving 158 records for screening. Title and abstract screening and full-text eligibility assessment were independently performed by two reviewers. Any disagreements were resolved through discussion and consensus.

In line with PRISMA-ScR methodology, no formal risk-of-bias or methodological quality assessment was conducted, as this scoping review aimed to map the breadth of existing evidence rather than evaluate study quality.

A total of 79 studies met the inclusion criteria and were included in the qualitative synthesis. To improve transparency, all included studies were summarized in a structured evidence table (**Supplementary Table S1)** and classified into clinical and preclinical (in vitro/in vivo) studies. The complete study selection process, including reasons for exclusion, is illustrated in the PRISMA-ScR flow diagram (**Supplementary Document S1**).

## Mechanism and application of PDT

PDT is initiated when a PS absorbs a photon of the light source. and generate ROS such as superoxide, hydrogen peroxide, and hydroxyl radicals. PS transfers energy directly to molecular oxygen (³O₂), producing singlet oxygen (¹O₂), which is typically the dominant cytotoxic species in PDT but depends on oxygen availability [22,23]. The resulting ROS oxidize lipids, proteins, and nucleic acids, triggering cell death pathways and promoting immunogenic signaling [22].

### Photosensitizers

PS are light-activatable molecules that, after administration and selective accumulation in target tissue, enable spatially controlled cytotoxicity upon irradiation [9,24]. Their therapeutic performance depends on intrinsic photophysical properties, pharmacokinetic behavior and tumor selectivity. Following systemic or topical delivery, PS distribution is influenced by tumor-specific features including abnormal vasculature, increased permeability, altered metabolism, association with endogenous plasma carriers (e.g., albumin and low-density lipoproteins (LDL)), and impaired lymphatic drainage, collectively resembling an enhanced permeability and retention (EPR)-like effect [6,[25], [26], [27]]. PS-generated ROS induce irreversible damage in microvasculature in endothelial cells and in vascular basement membrane. This is followed by platelet aggregation, release of vasoactive substances, leukocyte adhesion, which increases vascular permeability. The induced vascular collapse leads to tumor destruction [28].

Several PS have been evaluated for PDT, spanning first- and second-generation compounds with distinct photophysical characteristics, pharmacokinetics, and clinical outcomes [8,23]. From a therapeutic perspective, classification into generations remains useful because it reflects improvements in photodynamic efficiency, tissue penetration, and pharmacokinetics [6,29]. However, generational boundaries are not always strict, particularly for PS used as scaffolds for nanomedicine constructs. In HNSCC the most extensively investigated agents include first-generation porphyrin derivatives such as porfimer sodium (Photofrin®), second-generation chlorin- and bacteriochlorin-based PS such as temoporfin (Meso-tetrahydroxyphenyl chlorin, mTHPC) and 3-(1′-hexyloxyethyl) pyropheophorbide-a (HPPH), as well as prodrug-based systems such as 5-aminolevulinic acid (ALA), which relies on intracellular conversion to protoporphyrin IX (PpIX) [6,7,24]. These agents are activated by red to near-infrared (NIR) light (∼630–665 nm), a spectral window that enables relatively deep tissue penetration while maintaining efficient singlet oxygen production [30]. Most PDT applications in HNC have focused on HNSCC, the predominant histological subtype, although selected studies have also addressed premalignant lesions and, more rarely, other head and neck malignancies.

#### Classical PS

##### First-generation PS

Porfimer sodium represents the most extensively studied first-generation PS and provided early clinical evidence that PDT can achieve local tumor control and organ preservation in selected HNSCC patients. Its application has been particularly explored in early-stage lesions of the oral cavity, larynx, and pharynx, where PDT offers a minimally invasive alternative in anatomically complex sites [7,31]. However, porfimer sodium is characterized by prolonged cutaneous photosensitivity, relatively low molar absorptivity, limited tissue penetration at 630 nm, and non-specific tissue distribution, restricting its use in thicker or deeply infiltrative tumors and reducing patient acceptance [6,31].

##### Second-generation PS

Second-generation PS were developed to address the limitations of first-generation compounds by improving photodynamic efficiency, reducing systemic toxicity, and shifting absorption to longer wavelengths. In HNC, chlorin-based PS such as temoporfin (Foscan®), HPPH, and chlorin e6 (Ce6) have been most intensively investigated, with Foscan® remaining the only PS approved by the European Medicines Agency (EMA) for the treatment of early HNSCC and palliative treatment [7].

Temoporfin (mTHPC) exhibits strong absorption at approximately 652 nm, allowing deeper tissue penetration and markedly higher phototoxicity compared with porfimer sodium. Multi-institutional studies have demonstrated high local control and complete response (CR) rates in early-stage oral and oropharyngeal squamous cell carcinoma (SCC), with oncologic outcomes comparable to conventional therapies in selected patients [[32], [33], [34], [35]]. Nevertheless, mTHPC is associated with pronounced local phototoxicity and prolonged photosensitivity, which can be particularly problematic in anatomically delicate head and neck regions.

HPPH represents a further refinement within the chlorin class, combining longer-wavelength activation, reduced photosensitivity, and faster clearance than first-generation porphyrins [6,36]. Early clinical studies in oral SCC (OSCC) reported effective tumor ablation with improved tolerability [11,36]. However, recent analyses highlight that, similar to other classical PS, HPPH efficacy remains constrained by heterogeneous intratumoral distribution and oxygen dependence, limiting activity in hypoxic or stromal-rich tumors [37,38].

Ce6 exhibits strong red absorption (∼660–670 nm), high singlet oxygen yield, and faster clearance than earlier PS [39,40]. Preliminary clinical evidence has reported promising epithelial responses with limited toxicity in premalignant and dysplastic oral lesions [41,42]. Moreover, in selected patients with OSCC, preliminary clinical and long-term follow-up studies have reported encouraging local tumor control and acceptable tolerability [43,44]. Ce6-PDT has also been explored in combination with chemotherapy in a limited clinical experience, suggesting potential synergistic therapies that merit further investigation [45]. Furthermore, derivatives such as talaporfin sodium (mono-l-aspartyl chlorin e6, NPe6) have shown improved pharmacokinetic properties and reduced photosensitivity in small oral SCC cohorts [46].

##### Prodrug-based PS

ALA represents a distinct prodrug-based photodynamic strategy rather than a conventional second-generation PS. Following topical or systemic administration, ALA is metabolized via the heme biosynthetic pathway, leading to intracellular accumulation of PpIX, the active photosensitizing compound [7]. Preferential PpIX accumulation within transformed cells contributes to the tumor selectivity of ALA-PDT while minimizing prolonged systemic photosensitivity [47]. In head and neck oncology, ALA-PDT has been most extensively investigated in premalignant oral lesions, particularly oral leukoplakia and epithelial dysplasia, where clinical studies and systematic reviews reported favorable response rates with acceptable toxicity profile [[48], [49], [50]]. Applications in malignant HNSCC have been more limited and mainly focused on superficial early-stage oral lesions, including tongue and lip SCC [48,51]. Additionally, a pilot study combining ALA-PDT with chemotherapy suggested potential additive therapeutic effects [52].

#### Limitations of conventional PDT

Despite generational refinements, PDT in HNSCC continues to face persistent structural and biological barriers. These include restricted tissue penetration of activating light, suboptimal tumor selectivity, prolonged skin photosensitivity for certain agents, and reduced efficacy in hypoxic tumor regions [31,53]. Furthermore, robust prospective trials in advanced or aggressive HNSCC remain limited, and several PDT approaches still require repeated applications and may cause procedure-associated pain, particularly in superficial mucosal lesions. Consequently, conventional PDT remains primarily positioned for premalignant and early-stage disease [31,48]. A comparative overview of clinically investigated PS in HNSCC (Table 1) is therefore essential to contextualize outcomes and highlight the rationale for next-generation developments.

**Table 1:** An overview of key clinical studies evaluating PDT on HNSCC.

Photosensitiser (PS)	Study	Clinical indication	Study design / level of evidence (n)	PS dose & Light parameters	Main outcomes	Safety, toxicity and key limitations
1st generation						

**Photofrin** (porfimer sodium)	Biel, 1995 [54]	CiS, early stage (T1, T2), or palliative late stage (T2, T3) HNSCC	Single-centre case series; III (n = 60)	2 mg/kg | 630 nm | 50–200 J/cm^2^ | 24–60 h	- CiS and early T1, T2: 100% CR at 1 mo, <10% recurrence (25 mo mean). - Late T2, T3: 100% CR at 1 mo, ∼70% recurrence (<3 mo). - Stage IV: 4/4 PR at 1 mo, which recurred (<3 mo).	Highest efficacy in early-stage lesions. Prolonged photosensitivity and shallow penetration. Side effects such as pain and edema reported. Extensive clinical experience over years, particularly from Biel’s studies.
	Schweitzer and Somers, 2010 [55]	CiS, early stage or recurrent (T1) larynx SCC	Single-centre retrospective case series; III (n = 26)		- CiS: 50% CR with no recurrence (>2 mo); 50% PR which recurred (>2 mo). T1: 7/8 CR with no recurrence (>1 y); 1/8 PR which recurred (2 mo).	
	Hosokawa et al., 2020 [12]	T1, T2, T3 HNSCC	Single-centre retrospective case series; III (n = 42)		T1: ∼78% CR at 3 mo, <10% recurrence (<6 mo). T2: ∼60% CR at 3 mo, ∼40% recurrence (<6 mo). T3: 1/1 no response at 3 mo.	
	Narahara et al., 2023 [46]	Dysplasia and early stage (T1, T2) OSCC	Single-centre case series; III (n = 18)		Dysplasia and T1: ∼72% CR at 1 mo, <10% recurrence (>6 mo). T2: ∼71% CR at 1 mo, with ∼80% recurrence (>2 y).	

2nd generation						

**Temoporfin** (Foscan, mTHPC)	Hopper, et al., 2004 [32]	Early stage (T1, T2), or CiS OSCC	International multicentre Phase IIb trial; II (n = 121)	0.15 mg/kg | 652 nm |10–30 J/cm^2^ | 96 h	CiS: 3/3 CR T1: ∼90% CR T2: ∼58% CR. Recurrence, progression or metastasis: <10% (1 y); ∼10% (2 y).	Generally, well tolerated. Photosensitivity reactions observed in ∼10–20% of patients; rare severe adverse events reported (e.g., meningitis, visual impairments, tissue necrosis). Studies also include non-primary tumor settings.
	D'Cruz et al., 2004 [35]	Recurrent or refractory HNSCC	International multicentre study; II (n = 128)		∼16% CR at 1 mo, ∼55% longer survival	
	Karakullukcu et al., 2010 [34]	Early stage (T1, T2), or CiS, oral cavity and oropharynx SCC	International multicentre retrospective study; II (n = 170)		CiS: ∼80% CR. T1: ∼69% CR. T2: ∼59% CR. Recurrence: ∼25% (5 y).	
	Van Doeveren et al., 2018 [33]	Early (T1, T2), or late (T3, T4) stages, or CiS HNSCC	Single-centre case series; III (n = 54)		∼58% CR at 3 mo. Recurrence or progression: ∼42% (<3 mo); ∼63% (>3 mo).	

**HPPH**	Rigual et al., 2013 [36]	Early stage (T1), or CiS OSCC	Single-centre Phase I trial; III (n = 30)	4 mg/m^2^ | 665 nm | 50–140 J/cm^2^ (dose escalation) | 22–26 h	Dysplasia and CiS: ∼46% CR at 3mo, ∼66% (<9 mo). T1: ∼82% CR after 3 mo, ∼29% recurrence (<5 mo).	Generally, well tolerated. Pain and minor skin photosensitivity reported (e.g., erythema, edema, and necrosis).
	Shafirstein et al., 2015 [11]	Early stage (T1), or CiS larynx SCC	Single-centre Phase Ib trial; III (n = 29)		Dysplasia and CiS: ∼63% CR at 3 mo. T1: ∼82% CR after 3 mo, ∼10% recurrence (1 y), ∼20% (2 y).	

**Chlorin e6 & derivatives** (talaporfin sodium)	Büntzel et al., 2019 [44]	HNSCC (Palliative therapy)	Single-centre retrospective case series; III (n = 35)	Ce6: 1 mg/kg | 662–670 nm | 300 J/cm^2^ | 3 h	∼40% CR and ∼29% PR at 5 mo. No recurrence reported.	Favorable safety profile. Effective local control in palliative settings. Comparable outcomes to other PS.
	Panaseykin et al., 202,4^43^	Early stage (T1, T2) OSCC	Single-centre case series; III (n = 38)		∼92% CR at 3 mo, <10% recurrence (1–4 y).	
	Efendiev et al., 2025 [45]	T2, T3 OSCC	Phase I exploratory cases report; IV (n = 2)	Ce6: 0.5 mg/kg | 663 ± 5 nm | 80–150 J/cm^2^ | <1 h + chemotherapy	CR: 2/2 at 2 mo.	Relatively safe and feasible applicability. Intra-arterial administration. Limited generalizability.
	Narahara et al., 2023 [46]	Dysplasia and early stage (T1, T2) OSCC	Single-centre case series; IV (n = 5)	NPe6: 40 mg/m^2^ | 664 nm | 100 J/cm^2^ | 4 h	Dysplasia and T1: 3/3 CR at 1 mo, 1 recurrence (>9 m). T2: 2/2 CR at 1 mo, 1 recurrence (>6 m).	Favorable safety profile with reduced photosensitivity. Effective in early-stage OSCC.

**ALA/ PpIX**	Fan et al., 1996 [48]	Moderate/ severe dysplasia or refractory/ recurrent OSCC	Single-centre case series; III (n = 18)	60 mg/kg | 628 nm | 50–200 J/cm^2^ | fractionated 2.5 & 4 h or 4–6 h	Dysplasia: ∼91% CR, <10% (>1 y); OSCC: ∼50% CR, ∼33% recurrence (>1 y).	Generally well tolerated, with preferred topical administration. Mild to severe side effects reported (e.g., nausea, vomiting, pain, edema, mucositis, abnormal liver function). Highly heterogeneous applications (premalignant lesions, early SCC, and combination therapies), limiting direct comparison.
	Ahn et al., 2017 [51]	High-grade dysplasia, CiS, or early stage HNSCC	Single-centre Phase I study; III (n = 29)		CR: ∼69% at 3 mo. Recurrence: ∼48% (30 mo median)	
	Wang et al., 2021 [52]	Untreated OSCC (T1, T2, T3)	Retrospective case series; III (n = 11)	10% ALA cream | 635 nm | 100 J/cm^2^ | 2 h + chemotherapy (4 cycles)	T1: 1/1 CR at 1 mo. T2: 4/5 CR at 1 mo. T3: 2/5 CR at 1 mo. Recurrence: none (34 mo median)	

3rd generation						

**Redaporfin**	Lara Santos et al., 2018 [56]	T4 OSCC	Phase I/IIa exploratory case report; IV (n = 1)	0.75 mg/kg | 749 nm | 50 J/cm^2^ | ∼0 h + immunotherapy	CR: 1/1 at 1 and 3 mo.	Intravenous administration. Proof-of-concept; limited generalizability. Skin photosensitivity reported.

**Legend.** Evidence levels were evaluated according to Vaktar et al., 2025 adapting the predominance of non-randomized PDT studies in HNC, where treatment is frequently used in palliative, recurrent, or organ-preserving settings [57]. PS dose and light parameters are presented as: PS dose | wavelength | fluence | drug–light interval (hours). Outcomes comprehend complete response (CR), partial response (PR), and recurrence percentage that depend on each study criteria. In short, CR is defined as the absence of visible lesion with or without histological confirmation, while PR is defined as the reduction of at least 50% in maximum diameter of affected area or visible tumors. These outcomes vary significantly depending on tumor stage and patient selection. Recurrence ratios are reported in months (mo) or years (y). PS dose, light parameters, outcomes, and key notes represent a synthesis of the included studies for each photosensitizer, reflecting broadly consistent protocols and results across studies. Abbreviations: CiS: carcinoma in situ; SCC: squamous cell carcinoma; OSCC: oral SCC; HNSCC: head and neck SCC.

#### Efforts to overcome the limitations: third-generation PS and nanomedicine

Building upon the clinical experience summarized above, recent research has focused on refining PDT at both the molecular and microenvironmental levels. Third-generation photodynamic strategies have evolved along two complementary directions: (i) engineering novel PS with optimized photophysical and biological properties, and (ii) functional redesign of established PS through targeting ligands, nanocarrier and other delivery systems, and microenvironment-responsive platforms. Unlike conventional formulations, which rely largely on passive accumulation and uniform light activation, third-generation platforms aim to achieve selective tumor engagement through receptor-mediated targeting, EPR-driven nanodelivery, or microenvironment-responsive activation mechanisms, approaches particularly relevant in HNSCC [9,38,58]. Importantly, many emerging methodologies integrate several of these principles simultaneously, combining multiple functionalities within a single therapeutic construct [59].

##### Advanced near-infrared PS

In addition to delivery and targeting strategies, next-generation PS with optimized photophysical properties are being developed to enhance PDT efficacy. Redaporfin (LUZ11), a bacteriochlorin-based PS activated at ∼749 nm, enables deeper tissue penetration within the NIR window and displays rapid vascular-targeted photodynamic activity. Preclinical studies have demonstrated potent tumor ablation primarily mediated through acute vascular shutdown, leading to secondary tumor cell death [60]. LUZ11 is being evaluated in a clinical trial at recruiting stage (ID: NCT02070432). Its potential synergy with immune checkpoint inhibitors enhances its attractiveness for future application in HNSCC [56]. Although clinical evidence remains limited, LUZ11 illustrates how optimized PS features may expand PDT applicability to deeper-located or aggressive lesions.

##### Molecularly targeted PS

A key advance in third-generation PDT is the conjugation of PS to tumor-targeting ligands, including monoclonal antibodies, peptides, and small-molecule receptor binders [61]. This is a significant improvement with reduction of unspecific PS accumulation in skin or any other unwanted organs and localisations.

In HNSCC, epidermal growth factor receptor (EGFR) overexpression has provided a particularly attractive target for receptor-mediated delivery strategies. Preclinical studies using in vitro systems, patient-derived tumor samples, and in vivo models reported improved tumor-to-normal tissue contrast, reduced off-target vascular damage, and enhanced anti-tumor efficacy compared with non-targeted PS [62,63]. Similar approaches have shown selective efficacy in treating salivary gland cancer cell lines, further supporting the relevance of EGFR-directed PDT across head and neck malignancies [64].

Building on the introduction, CAFs represent a relevant stromal component in HNSCC. Importantly, CAFs are involved in resistance mechanisms to radio(chemo)therapy and immunotherapy in HNSCC [2]. Hence CAFs became targets of third-generation photodynamic strategies directed against FAP. Antibody- or ligand-conjugated PS targeting FAP accumulate in CAF-rich tumor compartments, especially in patients who have exhausted all treatment options, where light activation induces localized ROS-mediated phototoxicity that disrupts stromal support without directly targeting malignant epithelial cells [65,66]. Although, direct evidence in HNSCC models is still lacking, the high abundance of FAP^+^ CAFs in this disease provides a strong biological rationale for further translational investigation. The biological and therapeutic implications of CAF targeting in PDT are discussed in greater detail below. For multiresistant, metastatic HNSCC cancer with much disseminating tumor cells FAP-targeted radionuclide therapy is more efficient than PDT [67]. In addition, the combination of radionuclide therapy with PDT based on FAP- targeting PS and radionuclide conjugates might be cutting-edge strategies for immunosuppressed resistant HNSCC allowing unmasking the tumors to the immune system [68,69].

##### Delivery-Enhanced PS

Alternative strategies have explored endogenous carrier proteins, such as albumin, to enhance PS pharmacokinetics and tumor accumulation. Albumin-binding PS are associated with circulating serum albumin, prolong systemic PS circulation and facilitate tumor uptake [70]. Preclinical studies have confirmed that albumin-binding PS can achieve increased intratumoral retention and improved photodynamic efficacy, including in HNC models (e.g., EB-Ppa) [25]. Protein-mediated delivery represents a simpler but optimised strategy to enhance tumor selectivity compared with more complex engineered nanoplatforms.

Nanocarrier-based delivery systems incorporate PS into nanoscale platforms, including liposomes, polymeric nanoparticles (NPs), or inorganic constructs, to refine biodistribution and cellular delivery by ameliorating pharmacokinetics, protecting the drug during systemic circulation, and regulating tumor accumulation and intracellular release [58,71]. Ce6 is frequently employed as scaffold in such formulations due to its amphiphilic structure, strong absorption around 660 nm, high singlet-oxygen yield, and chemical versatility that facilitates chemical conjugation [6,39,58]. These features enable stable loading into liposomal and polymeric systems and integration with targeting or responsive elements, explaining its widespread use in nanomedicine-based PDT research. In head and neck models, polymer–lipid hybrid NPs and PEGylated liposomal carriers have been developed and tested preclinically to improve PS stability, tumor localization, and photodynamic efficacy [[72], [73], [74]]. Despite substantial progress, NPs-mediated PDT has not yet reached dedicated clinical trials in HNC patients.

### Light sources

A key determinant of PDT efficacy is the light source, which governs PS activation and tissue penetration. As discussed above, the wavelength must correspond to the PS absorption spectrum, typically in the NIR region. In this spectral window, reduced scattering and lower absorption by endogenous chromophores enable deeper tissue penetration [6,75]. This is particularly important in HNSCC, where lesions often arise in anatomically complex and partially shielded sites, including the oral cavity, oropharynx, and larynx.

Key parameters of light delivery include energy fluence, fluence rate, and illumination duration, which influence ROS generation, oxygen consumption, and treatment outcome. Clinically and experimentally used light sources include dye and diode lasers, light-emitting diodes (LEDs), and lamps [76]. Lasers provide high-intensity, single-wavelength light and allow precise delivery through fiber-optic or endoscopic systems, which is advantageous in the complex anatomy of the head and neck [76].

Continuous-wave (CW) lasers remain the clinical standard because they provide predictable dosimetry, stable PS excitation, and relatively low equipment costs. However, sustained illumination can rapidly deplete oxygen, which may exacerbate tumor hypoxia and limit singlet oxygen production, the principal cytotoxic mediator of PDT [77,78]. Pulsed irradiation delivers high-intensity bursts separated by dark intervals that allow partial oxygen replenishment. Experimental studies indicate that pulsed irradiation can reduce oxygen depletion and photobleaching while enhancing singlet oxygen generation and apoptosis at matched fluences [78,79]. Consistently, Rausch et al. demonstrated that these biochemical advantages are particularly relevant in higher-grade SCC tumors, where oxygen limitation strongly influences PDT efficacy [80]. Nevertheless, direct clinical comparisons between pulsed and CW PDT in HNSCC remain scarce and CW illumination remains the clinical benchmark.

Beyond lasers, light-emitting diodes (LEDs) have gained attention in wound healing and cancer therapy [[81], [82], [83]]. Recently, high-power organic light-emitting diodes (OLEDs) were preclinically evaluated for PDT in oral SCC models [84]. In this study, OLED-PDT significantly inhibited tumor growth, reduced proliferation, and induced apoptosis via ROS-mediated cytotoxicity, demonstrating that the achieved optical power densities could overcome previous light-output constraints [84]. Conversely, compared with laser systems, OLED-PDT required longer irradiation times, reflecting lower photon flux and potentially reduced tissue penetration.

### Exploiting cell death plasticity to optimize PDT

PDT triggers multiple regulated cell death pathways across cancer types [85]. Initially described as necrosis, apoptosis or autophagy, PDT is now known to induce multiple regulated cell death pathways including ferroptosis, necroptosis, pyroptosis, and mitotic catastrophe [85,86]. Among these mechanisms, ICD is particularly promising, as it combines direct tumor cytotoxicity with activation of adaptive immunity through the release of DAMPs and DC-mediated T-cell activation.

PDT-induced necrosis is characterized by extensive lipid peroxidation, cellular swelling, loss of plasma membrane integrity, DNA fragmentation, and the passive release of intracellular contents, including enzymes, HSP and DAMPs. These signals promote acute inflammation and contribute to ICD by activating innate anti-tumor immune responses [75,87](Fig. 2). Necrosis is associated with high light doses, elevated PS concentrations, or poorly confined illumination, which may compromise selectivity and increase photodamage [88].

PS subcellular localization dictates sites of oxidative damage and cell death mechanisms. Mitochondrial or endoplasmic reticulum (ER) targeting disrupts organelle integrity, leading to loss of mitochondrial membrane potential, cytochrome c release, activation of intrinsic apoptosis and DAMP emission. Lysosomal accumulation triggers membrane permeabilization, releasing cathepsins and hydrolases that induce necrosis or autophagy pathways [88,89], whereas plasma membrane localization primarily compromises membrane integrity [90,91].

Recent strategies aim to enhance PDT efficacy by targeting multiple organelles simultaneously. Wang et al. synthesized a curcumin-derived PS, with dual-organelle targeting in tumor cells, namely lipid droplets and ER [92]. Despite a low singlet oxygen quantum yield, this PS elicited potent photodynamic effects via complementary mechanisms: ER damage induced apoptosis, while ROS-mediated oxidation of lipid droplet polyunsaturated fatty acids triggered ferroptosis. Multi-organelle targeting, particularly of mitochondria and lysosomes, can enhance photodamage in large tumors by simultaneously activating multiple cell death pathways [93,94]. These approaches may employ single- or multi-wavelength activation schemes, although the latter increases technical complexity.

### Hypoxia as a central determinant of PDT efficacy and resistance

Multiple strategies have been developed to overcome tumor hypoxia within the TME. Mitochondria-localized PS can preserve cytotoxic activity under hypoxic conditions by favoring Type I photochemical reactions and by directly impairing oxidative phosphorylation, thereby reducing cellular oxygen consumption [95]. Additional approaches include in situ oxygen generation, nanotechnology-based oxygen delivery, and metabolic reprogramming. For example, Luo et al. engineered a tumor-targeted hybrid protein-based oxygen carrier that improves intratumoral oxygenation and enhances both chemotherapy and PDT efficacy [96].

Hypoxia-targeting nanoparticles integrating Ce6 with an anti-low-density lipoprotein receptor (LDLR) antibody demonstrated improved therapeutic outcomes in experimental HNSCC models [97]. Similarly, nanoformulations combining Ce6 with hypoxia-modulating agents, such as genistein-based systems, enhanced tumor suppression in nasopharyngeal carcinoma in vivo models by remodelling hypoxia-related pathways [98]. Other emerging strategies include ferroptosis-inducing nanodrugs and biomimetic nanoparticles, which amplify lipid peroxidation and ROS accumulation, thereby partially bypassing oxygen dependency [99,100]. Despite promising preclinical outcomes, these TME-responsive nanoplatforms have not yet entered clinical trials in HNSCC, highlighting a substantial translational gap.

Metabolic reprogramming has also emerged as an indirect strategy to modulate tumor oxygen dynamics. Inhibition of mitochondrial respiration using agents such as metformin has been investigated to decrease oxygen consumption and thereby improve oxygen availability for PDT [101,102]. In HNSCC in vitro models, combining metformin with laser irradiation was associated with increased phototoxicity and decreased AMPK-mTOR signaling, suggesting a synergistic effect beyond metformin monotherapy [102]. However, the translational relevance of these findings remains limited by simplified in vitro models and undefined PS protocols.

Hypoxia-inducible factor-1α (HIF-1α) represents a central regulator linking hypoxia, metabolism, immune evasion, and therapy resistance. Importantly, HIF-1α stabilization is not exclusively driven by hypoxia but can also be induced by oncogenic signaling, inflammatory pathways, and metabolic stress [103,104]. Notably, PDT itself may induce HIF-1α expression through ROS-mediated signaling, potentially activating adaptive survival pathways and limiting therapeutic efficacy [105]. Consistently, Lamberti et al. identified a ROS/ERK/HIF-1α signaling axis as an intrinsic mechanism of PDT resistance, highlighting hypoxia-independent HIF-1α activation as a promising therapeutic target [106].

## Improving anti-tumor immunity with PDT

When treating superficially spreading tumors with flat light sources or deeply located tumors using interstitial illumination (e.g. needles or cannulas), complete tumor coverage by PDT remains challenging. Consequently, clinical use was considered mainly for smaller and localised tumors, that can be effectively illuminated. Although such lesions are often suitable for surgical resection, combining surgery with PDT may provide additional benefits by targeting residual malignant cells and circulating tumor cells (CTCs) potentially involved in tumor dissemination [107]. Achieving this effect requires careful optimization of PS dose, PDT scheduling, and the generation of DAMPs, together with a mechanistic understanding of DC, T-cell, and natural killer (NK) cell responses. Interestingly, HNSCC in vivo studies demonstrated that lower PDT doses may induce stronger immune activation than higher doses [108]. Nevertheless, PDT-induced apoptosis and ER stress remain critical triggers of the induction of ICD [87]. Following low-dose PDT, dying tumor cells release DAMPs that promote DC maturation and antigen presentation, thereby initiating adaptive immune responses characterized by the activation of NK cells and CD8+ T cells in HNSCC models [109,110](Fig. 2).

Combining PDT-mediated tumor cell killing with immune priming and radiotherapy represents a promising preclinical strategy with potential clinical relevance. Experimental work suggested that T cell activation and alterations to the tumor bed, meaning PDT-based tumor cell killing, radiotherapy, and generation of DAMPs, must occur simultaneously [87,110]. Korbelik et al. further investigated this path by describing HNSCC vaccines generated by PDT treatment. The authors used mouse models and demonstrated that tumor cells derived from surgically resected tumors, treated with chlorin e6–mediated PDT and incubated for 16 h post-treatment, could serve as effective tumor cell-based vaccines [110].

## Targeting CAFs with PDT

CAFs may survive cytotoxic therapies more effectively than tumor cells and support the regrowth of residual tumor cells, thereby contributing to local tumor recurrence [111,112].

Biochemically, CAF-derived TGF-β induces a slow-cycling state in tumor cells, reducing their susceptibility to ROS-mediated PDT. Inhibition of TGF-β signaling has been shown to reverse this resistance in cutaneous SCC models [113]. CAFs remodel the TME through collagen deposition and ECM stiffening, which impairs PS penetration, oxygen and light diffusion within the tumor tissue [3,114]. These stromal alterations also substantially limit the therapeutic efficacy of non-targeted PDT.

In this direction, conventional PS-based PDT exerts indirect CAF-modulating effects, as shown for ALA treatments. In SCC–CAF co-culture setups, ALA-PDT reduced CAFs activation, as indicated by decreased expression of α-smooth muscle actin (α-SMA), FAP, and collagen, while simultaneously inducing ROS-mediated CAF apoptosis and ECM remodelling [115]. These changes facilitate immune cell infiltration, supporting the concept that CAF normalization, rather than complete depletion, may improve PDT outcomes (Fig. 2).

Building on these observations, nanodrug-based strategies could be implemented to selectively target and reprogram active myofibroblastic CAFs toward a quiescent state [116]. Following CAF normalization, reduced collagen deposition and improved vascular and immune function within the TME are expected to ultimately enhance PDT efficacy [115].

## Barriers to clinical translation of PDT in HNSCC

Although newest-generation PS, targeting strategies, and immune-mediated anti-tumor effects continuously reach clinical application, the clinical studies are small and isolated. The penetrance and systematic skin sensitivity were biological and technical limitations which are continuously improved. Regulatory and economic factors, further hinder the clinical integration of advanced PDT platforms [117,118].

Nanoparticle- and antibody-based systems often require complex manufacturing processes, extensive quality control measures, and dedicated device integration, resulting in increasing costs and limiting scalability. In addition, reimbursement pathways for PDT in HNSCC remain insufficiently established in many healthcare systems, particularly for multifunctional combination products that do not readily fit within conventional drug frameworks [117]. These challenges likely contribute to the limited number of clinically approved targeted PDT approaches despite encouraging preclinical findings.

Collectively, these findings highlight the considerable gap between the promising biological rationale of PDT and its current clinical impact in HNSCC. Anatomical complexity, hypoxia, stromal barriers, and heterogeneous PS distribution continue to compromise therapeutic efficacy. In addition, translational challenges associated with targeted delivery systems and combination strategies with immunotherapy further complicate therapeutic use.

An additional consideration is that clinical guidelines require strong evidence for modification. Accordingly, PDT is likely to be considered within multimodal treatment strategies, potentially including after neoadjuvant immunotherapy, surgical resection, and after combined standard-of-care regimens. This underscores the need for PDT development to address therapy-resistant tumor cell populations contributing to recurrence and metastasis. A basically local treatment approach may therefore be insufficient. A potentially relevant clinical niche may exist in patients with unresectable tumors who are not eligible for standard neoadjuvant immunotherapy protocols. In this setting, there may still be potential for immune-mediated tumor cell killing, which could be boosted by PDT-induced ICD, potentially defining a future role for PDT in HNSCC [119].

## Conclusion

PDT represents a promising multimodal strategy in HNSCC**,** inducing tumor cytotoxicity and ICD while sparing surrounding tissue. Limitations of conventional PS, including hypoxia, stromal stiffness, and limited light penetration, have spurred development of nanoparticle-based strategies. CAFs survive therapies and effectively support tumor regrowth and progression. For this reason, CAF-targeting PDT enables remodelling of the ECM, improves PS delivery and enhances systemic anti-tumor immune responses. Continued translational and clinical research is needed to optimize PDT protocols, refine stromal targeting, and integrate immunotherapeutic combinations to improve outcomes in HNSCC.

## Abbreviations

The following abbreviations are used in this manuscript:

ATP – Adenosine triphosphate.

CAFs – Cancer-associated fibroblasts.

CAF-derived exosomes – CAF-Exos.

CD – Cluster of differentiation.

CR – Complete response.

CW – Continuous wave.

CXCL12 – C-X-C motif chemokine ligand 12.

DAMPs – Damage-associated molecular patterns.

DCs– Dendritic cells.

ECM – Extracellular matrix.

ER – Endoplasmic reticulum.

EGFR – epidermal growth factor receptor.

FAP – Fibroblast activation protein.

HNSCC – Head and neck squamous cell sarcinoma.

HNC – Head and neck cancer.

HIF-1α – Hypoxia-inducible factor 1-alpha.

HMGB1 – High mobility group box 1.

HSP – Heat shock proteins.

ICD – Immunogenic cell death.

IL-6 – Interleukin-6.

MDSCs – Myeloid-derived suppressor cells.

OLEDs – Organic light-emitting diodes.

OSCC – Oral Squamous Cell Carcinoma.

PD-L1 – Programmed death-ligand 1.

PR – Partial response.

PDT – Photodynamic therapy.

PS – Photosensitizer.

SCC – squamous cell carcinoma.

TGF-β – Transforming growth factor-beta.

During the preparation of this work the authors used ChatGPT for grammar checking and language editing. After using this tool, the authors reviewed and edited the content as needed and take full responsibility for the content of the publication.

## Institutional review board statement

Not applicable.

## Informed consent statement

Not applicable.

## Funding

This work was funded by the 10.13039/501100002428Austrian Science Funds (FWF), project number: I 3976-B33. Grant DOI: 10.55776/I3976.

## CRediT authorship contribution statement

**Julia Federspiel:** Writing – review & editing, Writing – original draft, Investigation, Conceptualization. **Federico Ferro:** Writing – review & editing, Writing – original draft, Investigation. **Jozsef Dudas:** Writing – review & editing, Supervision, Funding acquisition. **Anna Katharina Stenzl:** Writing – review & editing. **Benedikt Gabriel Hofauer:** Writing – review & editing, Supervision.

## Declaration of competing interest

The authors declare that they have no known competing financial interests or personal relationships that could have appeared to influence the work reported in this paper.

## Data Availability

No new data were created or analyzed in this study.

## Appendix Supplementary materials

Image, application 1

Image, application 2
