# Supplementary material for: Photodynamic therapy in head and neck squamous cell carcinoma: immunomodulation and stromal targeting of cancer-associated fibroblasts
Source: Transl Oncol. 2026 Jul 9;71:102910. doi: 10.1016/j.tranon.2026.102910 (PMC13380419; doi:10.1016/j.tranon.2026.102910)
Supplement: Supplementary file 1 [file mmc1.docx]

**Supplementary Document S1: Study selection process according to the PRISMA-ScR framework.**

**Identification**

Duplicates removed (n=34)

Records identified through database searching (n=192)

Records after duplicates removed (n=158)

**Screening**

Records removed (n= 44)

Records screened by title and abstract (n=158)

Full-text articles excluded with reasons* (n=35)

Full-text articles assessed for eligibility (n=114)

**Eligibility**

Studies included in qualitative synthesis (n=79)

Total references cited (n= 120)

**Included**

* not related to PDT, no CAF/TME relevance, not related to head and neck oncology, not tumor-related, editorials, conference abstracts, non-English article, insufficient mechanistic data.
