# Supplementary material for: Photodynamic therapy in head and neck squamous cell carcinoma: immunomodulation and stromal targeting of cancer-associated fibroblasts
Source: Transl Oncol. 2026 Jul 9;71:102910. doi: 10.1016/j.tranon.2026.102910 (PMC13380419; doi:10.1016/j.tranon.2026.102910)
Supplement: Supplementary file 2 [file mmc2.docx]

**Overview of all included original research studies (n=79).**

| **No.** | **Year** | **Author** | **Study type** | **Key findings** |
| --- | --- | --- | --- | --- |
| 1 | 1993 | \|  \| \| --- \|   Rausch et al. | Preclinical (in vivo) | pulsed irradiation improves PDT efficacy compared to continuous wave |
| 2 | 1995 | Biel | Clinical | early clinical PDT applications demonstrate feasibility for head and neck malignancies |
| 3 | 1996 | Fan et al. | Clinical | ALA-PDT shows clinical efficacy in premalignant and malignant oral cavity lesions |
| 4 | 1996 | \|  \| \| --- \|   Cincotta et al. | Preclinical (in vivo) | combination PDT induces complete regression of large murine sarcomas |
| 5 | 1999 | Miyamoto et al. | Preclinical (in vitro) | pulsed and continuous-wave irradiation differ in phototoxicity mechanisms in PDT-treated cells. |
| 6 | 2004 | Hopper et al. | Clinical | mTHPC-PDT, clinical efficacy in early oral SCC with high local tumor response rates. |
| 7 | 2004 | D’Cruz et al. | Clinical | mTHPC-PDT, tumor responses and palliative benefit in advanced HNSCC |
| 8 | 2004 | Kawauchi et al. | Preclinical (in vitro) | PDT with pulsed vs continuous-wave laser alters oxygen consumption in tumor cells |
| 9 | 2007 | Korbelik et al. | Preclinical (in vivo) | PDT-induced tumor cell death can generate vaccine-like anti-tumor immune responses |
| 10 | 2009 | Yu et al. | Preclinical (in vitro) | PDT efficacy varies with light source |
| 11 | 2009 | Stasinopoulos et al. | Preclinical (in vitro) | inflammatory signaling contributes to HIF-1α activation in tumor cells under PDT |
| 12 | 2010 | Karakullukcu et al. | Clinical retrospective | PDT achieves local tumor control in early oral/oropharyngeal tumors |
| 13 | 2011 | \| Ali-Seyed et al. \| \| --- \| | Preclinical (in vitro) | PDT induces ROS-mediated apoptosis via mitochondria–lysosome crosstalk |
| 14 | 2011 | Schweitzer & Somers | Clinical | PDT with Photofrin is effective in early-stage laryngeal tumors |
| 15 | 2012 | Schartinger et al. | Preclinical (in vitro) | LLLT induces differential responses in fibroblasts and tumor cells |
| 16 | 2012 | Yano et al. | Clinical | salvage PDT provides local tumor control in recurrent esophageal squamous cell carcinoma after chemoradiotherapy |
| 17 | 2013 | Sobaniec et al. | Clinical | PDT shows clinical efficacy in the treatment of oral lichen planus with symptom improvement |
| 18 | 2013 | Rigual et al. | Clinical | HPPH-mediated PDT shows clinical efficacy in oral cavity cancer with tumor response and manageable toxicity. |
| 19 | 2014 | Pietruska et al. | Clinical | PDT, oral leukoplakia, lesion reduction, clinical efficacy |
| 20 | 2015 | Shafirstein et al. | Clinical | PDT, HPPH, early laryngeal cancer, phase Ib, safety, tumor response |
| 21 | 2016 | Ahn et al. | Clinical | phase I PDT trial shows acceptable toxicity and early clinical responses in premalignant and early-stage head and neck tumors |
| 22 | 2016 | Lv et al. | Preclinical (in vivo + in vitro) | mitochondria-targeted photosensitizer enhances PDT efficacy under hypoxic conditions |
| 23 | 2016 | \| Klimenko et al. \| \| --- \|  \|  \| \| --- \| | Preclinical (in vitro) | pulsed laser PDT induces apoptosis in cancer cells via enhanced photodynamic efficiency |
| 24 | 2017 | Theodoraki et al. | Clinical | PDT modulates peripheral immune cell populations and cytokine levels in head and neck cancer patients |
| 25 | 2017 | \|  \| \| --- \|   Ma et al. | Preclinical (in vivo + in vitro) | PDT, nanoparticle siRNA delivery, EMT inhibition, enhanced tumor sensitivity |
| 26 | 2017 | Lamberti et al. | Preclinical (in vitro) | ROS–ERK–HIF-1α signaling mediates resistance to photodynamic therapy in cancer cells |
| 27 | 2017 | Pramual et al. | Preclinical (in vitro) | polymer-lipid-PEG hybrid nanoparticles improve photosensitizer delivery and PDT efficiency |
| 28 | 2018 | Hosokawa et al. | Clinical | PDT shows clinical efficacy and feasibility in patients with head and neck squamous cell carcinoma |
| 29 | 2018 | van Doeveren et al. | Clinical | adjuvant PDT improves local tumor control after positive surgical margins in head and neck cancer |
| 30 | 2018 | Gomes-da-Silva et al. | Preclinical (in vitro) | Redaporfin, ROS-mediated ER/Golgi damage, ICD |
| 31 | 2018 | Santos et al. | Clinical | Redaporfin-PDT achieved complete local tumor ablation in a heavily pretreated head and neck cancer patient, followed by durable response after sequential immunotherapy |
| 32 | 2018 | Luo et al. | Preclinical (in vivo) | PDT shows enhanced tumor growth inhibition in vivo models |
| 33 | 2019 | Büntzel et al. | Clinical | Ce6-based PDT applied in palliative head and neck cancer treatment |
| 34 | 2019 | Driehuis et al. | Preclinical (ex vivo / in vitro) | patient-derived HNSCC organoids reproduce EGFR expression and show sensitivity to EGFR-targeted PDT |
| 35 | 2019 | Doix et al. | Preclinical (in vivo) | low-dose PDT combined with early radiotherapy enhances dendritic cell vaccine–mediated anti-tumor immunity |
| 36 | 2019 | Li S. et al. | Preclinical (in vitro) | ALA-PDT reverses CAF activation and modulates the TME |
| 37 | 2019 | Turubanova et al. | Preclinical (in vivo + in vitro) | PDT induces ICD and anti-tumor immune activation |
| 38 | 2019 | Ricco et al. | Preclinical (in vitro) | mevalonate pathway activity determines radiosensitivity in HNSCC |
| 39 | 2020 | Kieffer et al. | Preclinical (in vivo) | CAF subsets are associated with immunotherapy resistance and poor prognosis |
| 40 | 2020 | Song et al. | Preclinical (in vitro + vivo) | hypoxia-targeting nanoparticles enhance chemo- and phototherapy efficacy in HNSCC |
| 41 | 2020 | Peng et al. | Preclinical (in vivo) | EGFR-targeted PDT improves tumor selectivity and treatment efficacy |
| 42 | 2021 | Domogauer et al. | Preclinical (in vitro) | CAFs exhibit enhanced antioxidant and DNA repair capacity associated with radioresistance |
| 43 | 2021 | Cheraghlou et al. | Retrospective observational | PDT use and Medicare spending increased from 2012–2017, with persistent geographic access disparities in the United States |
| 44 | 2021 | Gallego-Rentero et al. | Preclinical (in vitro) | CAF-derived TGF-β1 induces resistance to PDT in squamous cell carcinoma cells |
| 45 | 2021 | Fischlechner et al. | Clinical | low-level laser therapy shows no survival benefit in HNSCC patients |
| 46 | 2021 | Lambert et al. | Clinical | PDT is a feasible alternative treatment in functionally inoperable oral and oropharyngeal carcinoma |
| 47 | 2021 | Wang X. et al. | Clinical | ALA-PDT combined with chemotherapy improves response in advanced oral SCC |
| 48 | 2021 | Turubanova et al. | Preclinical (in vivo + in vitro) | porphyrazine-based PDT induces ICD and anti-tumor immune activation |
| 49 | 2021 | Liu W. et al. | \|  \| \| --- \|   Preclinical (in vitro) | Ce6-biotin conjugates enable targeted delivery and enhanced cytotoxicity in cancer cells |
| 50 | 2022 | Obradovic et al. | Preclinical (in vivo, human tumor profiling) | CAF subpopulations predict immunotherapy response and shape tumor immune landscape |
| 51 | 2022 | Li S. et al. | Preclinical (in vivo) | PDT-based dendritic cell vaccine enhances anti-PD-L1 immunotherapy efficacy |
| 52 | 2022 | \|  \| \| --- \|   Tracy et al. | Preclinical (in vitro) | intracellular photosensitizer retention determines PDT efficacy in tumor cells |
| 53 | 2022 | Mishchenko et al. | Preclinical (in vivo + in vitro) | PDT-induced cell death modality depends on photosensitizer, dose, and irradiation parameters |
| 54 | 2022 | Shi et al. | Preclinical (in vitro) | LED-PDT induces ROS-dependent apoptosis and necrosis in cancer cells |
| 55 | 2022 | Zhang, Y. et al. | Preclinical (in vitro) | radiolabeled nanoparticle PDT enhances tumor cytotoxicity |
| 56 | 2023 | Minagawa et al. | Preclinical (in vitro) | LED-PDT induces ROS production and enhances cellular stress responses |
| 57 | 2023 | Dorst et al. | Preclinical (in vivo) | FAP-targeted PDT selectively depletes CAFs and reprogram the TME |
| 58 | 2023 | Fei et al. | \|  \| \| --- \|   Preclinical (in vivo) | CAF reprogramming + vascular normalization to enhance PDT |
| 59 | 2023 | Ebrahimi et al. | Preclinical (in vitro) | metformin + laser induces synergistic cytotoxicity in tumor cells |
| 60 | 2023 | Narahara et al. | Clinical | PDT provides long-term tumor control in oral squamous cell carcinoma and epithelial dysplasia |
| 61 | 2023 | \|  \| \| --- \|   Liu H. et al. | Preclinical (in vitro) | albumin-binding photosensitizer enables imaging-guided targeted PDT with enhanced tumor uptake |
| 62 | 2023 | \|  \| \| --- \|   Mou et al. | \|  \| \| --- \|   Preclinical (ex vivo) | CAF heterogeneity shapes tumor–immune interactions in HNSCC organoids |
| 63 | 2023 | \|  \| \| --- \|   Li Y. et al. | Preclinical  (in vitro) | lysosome-targeting nanophotosensitizer induces lysosomal membrane permeabilization and enhances PDT cytotoxicity |
| 64 | 2023 | \|  \| \| --- \|   Wang Y. et al | Preclinical  (in vitro) | dual organelle-targeting photosensitizer enhances ROS generation and cytotoxicity in cancer cells |
| 65 | 2024 | Glabman et al. | Preclinical (in vivo) | FAP-targeted therapy depletes CAFs and modulates the TME |
| 66 | 2024 | Sun et al. | Preclinical (in vivo) | PDT induces adaptive immune resistance via HIF-1α/PD-L1 upregulation |
| 67 | 2024 | Panaseykin et al. | Clinical | Ce6-PDT shows long-term tumor control in oral cavity squamous cell carcinoma |
| 68 | 2024 | Yamaguchi et al. | Preclinical (in vivo) | EGFR-targeted NIR photoimmunotherapy suppresses tumor growth in vivo in salivary gland cancer |
| 69 | 2024 | Wang Y. et al. | Clinical | ALA-PDT, oral SCC, clinical response, tolerability |
| 70 | 2025 | Chatterjee et al. | Preclinical  (in vitro) | serum albumin enhances delivery of NIR photosensitizer and increases cellular uptake in breast cancer cells |
| 71 | 2025 | Efendiev et al. | Clinical | intra-arterial Ce6/PDT combined with cisplatin shows first clinical efficacy signals in HNSCC |
| 72 | 2025 | Federspiel et al. | Preclinical (in vitro) | patient-derived CAFs promote tumor cell colonization in HNSCC |
| 73 | 2025 | \|  \| \| --- \|   Thibaudeau et al. | Preclinical (in vitro) | ER stress intensity inversely correlates with anti-tumor immune response (Ru(II) photosensitizers, HNSCC) |
| 74 | 2025 | \|  \| \| --- \|   Paul & Biswas | Preclinical (in vitro + in vivo) | NIR-responsive Ce6 prodrug micelles enable targeted PDT in oral cancer |
| 75 | 2025 | Carrizo et al. | Preclinical (in vitro + in vivo) | single-point laser PDT enables selective plasma damage and tumor cell death |
| 76 | 2025 | Zhou Q. et al. | Preclinical (in vitro) | e6/Genistein nanocarrier remodels hypoxic tumor microenvironment and enhances PDT efficacy in NPC |
| 77 | 2025 | Zhang J. et al. | Preclinical (in vivo + in vitro) | OLED-based light delivery improves PDT efficacy in OSCC mouse model |
| 78 | 2026 | Abdurashitov et al. | Preclinical (device/experimental bench study) | LED-based device enables standardized PDT irradiation |
| 79 | 2026 | Lu et al. | Preclinical (in vitro + in vivo) | biomimetic nanoplatform enhances ferroptosis–PDT synergy in oral tongue SCC |

**Abbreviations:** PDT: Photodynamic Therapy; HNSCC: Head and Neck Squamous Cell Carcinoma; SCC: Squamous Cell Carcinoma; OSCC: Oral Squamous Cell Carcinoma; NPC: Nasopharyngeal Carcinoma; LLLT: Low-Level Laser Therapy; ROS: Reactive Oxygen Species; ICD: Immunogenic Cell Death; TME: Tumor Microenvironment; CAF: Cancer-Associated Fibroblast; EMT: Epithelial–Mesenchymal Transition; HIF-1α: Hypoxia-Inducible Factor 1-alpha; EGFR: Epidermal Growth Factor Receptor; TGF-β1: Transforming Growth Factor beta 1; Ce6: Chlorin e6; HPPH: 3-(1′-hexyloxyethyl) pyropheophorbide-a; ALA: 5-aminolevulinic acid; mTHPC: meta-tetrahydroxyphenylchlorin; ER: Endoplasmic Reticulum;
